# Minimizing antibiotic exposure in the ICU: in search of the optimal strategy

**DOI:** 10.1186/s13054-014-0613-y

**Published:** 2014-10-31

**Authors:** Christian Brun-Buisson

**Affiliations:** Service de Réanimation Médicale, Hôpital Henri Mondor, Université Paris-Est Créteil, 51 Avenue du Maréchal de Lattre de Tassigny, 94010, Créteil, Cedex France

## Abstract

The current paradigm for antibiotic management in critically ill patients is to initiate broad-spectrum therapy followed by de-escalation based on microbiological results. Routine screening cultures may allow better targeting and reduce unnecessary exposure to antibiotics.

The primary goal of active surveillance cultures (ASC) is to guide preventive strategies to avoid transmission of multidrug-resistant bacteria, by identifying carriers and implementing or maintaining isolation and contact precautions when indicated. ASC may also be used to predict infection with multidrug-resistant bacteria in septic patients and better tailor antimicrobial therapy, especially for ventilator-associated pneumonia (VAP), as suggested in a previous issue of *Critical Care* by a group of investigators in Ghent [1].

De Bus and colleagues analyzed 113 episodes of hospital-acquired pneumonia (HAP), including 52 VAP episodes, to examine whether a strategy based on ASC performed better than a guideline-based strategy derived from the American Thoracic Society/Infectious Diseases Society of America guideline [2] and adapted to their local epidemiology.

There are three distinctive features of this study [1]. First, the authors derived the prescription algorithms subsequently tested from a previous analysis of 100 episodes of pneumonia; although few details are provided on their construction, the algorithms were intended to provide >85% adequate antimicrobial coverage while avoiding overtreating patients with unnecessarily broad-spectrum antibiotics.

Second, De Bus and colleagues compared both the appropriateness and spectrum of the actual antibiotic prescriptions with that of regimens that would have been administered according to one or the other algorithm-based strategies, using a scale to grade the spectrum of antibiotics prescribed, from narrower spectrum to broader spectrum.

Third, the authors included both HAP and VAP in similar proportions in their analysis, whereas most previous studies have focused on VAP [3-5] or nosocomial bloodstream infection [6,7]. However, the latter is both a strength and a weakness. Applying ASC to pneumonia in nonventilated patients is of potential interest; however, the microbiologic features of HAP may differ from those of VAP, and obtaining ASC in these patients (especially respiratory tract cultures) is more difficult. Indeed, recent (2 to 5 days) respiratory tract samples to target therapy were available in 63 (56%) of all 113 episodes and were positive in 43 (38%) cases; these samples would have guided therapy in 31/52 (60%) episodes of VAP, but in only 12/61 (20%) episodes of HAP [1].

Although the authors did not stratify their analysis according to HAP or VAP cases, it can be inferred that most HAP (non-VAP) episodes were included in the subgroup of the ASC-based strategy where positive ASC from the respiratory tract dating more than 5 days earlier or from other sites were available (*n* = 70), rather than from recent respiratory tract samples that are known to more accurately predict the etiology of subsequent pneumonia [8]. In this subgroup, positive samples were available for 28 (40%) episodes, which led to upgrading the antibiotic regimen in 13 (19%) cases and to increasing the appropriateness of therapy by 10% (from 79 to 88%). ASC therefore seemed to help narrow the spectrum in VAP episodes, but to broaden the spectrum in HAP (non-VAP) episodes.

It is noteworthy that De Bus and colleagues found no difference in the appropriateness of therapy when comparing the two (theoretical) algorithm-based strategies with the actual therapy received by patients [1]. However, treating physicians were aware of the results of ASC and were probably influenced to some extent by them, given the longstanding tradition of using ASC at this center [3,7,9].

Also apparent from this study is that the standard guideline-based approach to therapy, even when adapted to the local epidemiology, often results in broader-spectrum therapy than required. As discussed above, this may be due in large part to the high proportion of patients with HAP (non-VAP) in this study, consistent with a number of recent studies showing that the criteria for suspected infection with multidrug-resistant bacteria in patients with HAP or healthcare-associated pneumonia are nonspecific, at least when applied to European populations [10-12].

Given the higher proportion of appropriate and narrower-spectrum therapy with ASC than with the guideline-based approach, should ASC become routine practice in our ICUs? Clearly, a randomized trial – preferably a crossover cluster-randomized study – is now needed to answer this question. This trial would compare a strategy including ASC as described by De Bus and colleagues with the standard of care approach, based on empirical therapy according to the physician’s best judgment accounting for prior antibiotic exposure, local epidemiology and the patient’s individual risk factors, followed by de-escalation [5,13]. The trial should examine patient-centered outcomes, appropriateness of antibiotics, and overall antibiotic exposures. A formal cost-effectiveness of this approach is also needed, since routinely obtaining thrice-weekly urinary and sputum or endotracheal aspirate cultures in addition to weekly oral, nasal, and rectal swab cultures, as is performed in Ghent, bears substantial costs. Finally, routine surveillance respiratory or urine cultures may also incite physicians to initiate therapy for colonization rather than for infection, and eventually increase the – microbiologically appropriate, but unnecessary – antibiotic exposure of ICU patients.

## Abbreviations

ASC: Active surveillance cultures
HAP: Hospital-acquired pneumonia
VAP: ventilator-associated pneumonia
